# Clinical Characteristics and Risk Factors of *Clostridioides difficile* Infection: A Case–Control Study in a High-Complexity Clinic in Santiago de Cali, Colombia

**DOI:** 10.3390/jcm15062090

**Published:** 2026-03-10

**Authors:** Duvan Arley Galindes-Casanova, Edith Norela Benitez-Escobar, Jorge Enrique Daza-Arana, Heiler Lozada-Ramos, Juan Carlos Ávila-Valencia, José Millán Oñate-Gutiérrez

**Affiliations:** 1 Internal Medicine Specialization Program, Faculty of Health, Universidad Santiago de Cali, Cali 760035, Colombia; drdagasc2024@gmail.com (D.A.G.-C.); edithnorela2018@gmail.com (E.N.B.-E.); heiler.lozada00@usc.edu.co (H.L.-R.); millanonate@gmail.com (J.M.O.-G.); 2 Metabolism, Physiology, Genetics, and Research Group (GEFIME), Faculty of Health, Universidad Santiago de Cali, Cali 760035, Colombia; 3 Departament of Research and Education Department, Clínica de Occidente S.A., Cali 760035, Colombia; rhcardiopulmonar09@gmail.com

**Keywords:** *Clostridioides difficile*, risk factors, antibiotics, proton pump inhibitor, hospitalization

## Abstract

**Objectives:** This study aimed to describe the epidemiological and clinical characteristics and the potential risk factors associated with *Clostridioides difficile* infection (CDI) in a high-complexity healthcare center. **Methods:** This was a retrospective case–control study conducted from 2020 to 2022 with a cohort of participants aged ≥18 years with diarrhea (more than three liquid stools per day), which included a molecular testing request (the FilmArray Gastrointestinal [GI] PCR Panel) in a high-complexity clinic in Santiago de Cali, Colombia. Controls were randomly selected from the same institutional laboratory database at a 2:1 ratio, matched by age and sex, and required to test negative for *C. difficile*. Patients from other institutions were excluded to avoid exposure misclassification. **Results:** Our study included 147 participants (49 cases and 98 controls) and found a 22% infection prevalence among those who underwent molecular testing. When comparing CDI cases with controls, significant differences were observed in the univariate analysis: cases showed longer time to symptom resolution, longer post-diagnosis hospital stay, and greater exposure to in-hospital antibiotics for more than 7 days prior to symptom onset (*p* < 0.05). Among CDI cases, 55% were healthcare-associated and 18% were classified as severe, with an overall 30-day mortality of 15%. In the multivariate logistic regression model, three variables remained significantly associated with CDI: hospital stay longer than 10 days before symptom onset, antibiotic exposure in the previous 90 days, and in-hospital proton pump inhibitor use. **Conclusions:** CDI can present a wide range of clinical manifestations, so underdiagnosis should be avoided. Identifying risk factors, particularly in patients with hospital-acquired diarrhea, is crucial. Factors such as a hospital stay longer than 10 days before symptom onset and in-hospital exposure to PPIs or antibiotics in the last 90 days were significant in our study. Early recognition of these risk factors may reduce hospital stay, lower the risk of complications, and optimize healthcare resources.

## 1. Introduction

*Clostridioides difficile* (CD) is one of the main causes of infection associated with healthcare worldwide, particularly associated with diarrhea secondary to antibiotic use. *Clostridioides difficile* infection (CDI) affects morbidity and mortality, as well as health care costs [1]. Its epidemiological behavior varies considerably, and incidences ranging from 20 to >600 annual cases per every 100,000 inhabitants have been reported, with increasing reports of community-acquired cases and a risk of recurrence that can exceed 30% [2,3]. There is no international consensus on the exact timing for assessing parameters in relation to treatment initiation. The updated ESCMID guidelines incorporate new severity definitions, which are now more aligned with the IDSA/SHEA recommendations [4].

CD is a Gram-positive, spore-forming bacterium that can be transmitted through the fecal–oral route. Under certain environmental conditions and risk factors, it acquires its vegetative form and produces toxins which, in turn, trigger its corresponding pathological process and clinical manifestations [3]. Risk factors such as immune status, age, exposure to drugs such as antibiotics and proton pump inhibitors (PPIs), comorbidities such as chronic kidney disease, neoplasms, and the circulation of hypervirulent strains of the NAP-1/027 type result in a disease spectrum ranging from asymptomatic colonization to severe and fulminant disease associated with multiple complications [5,6]. It has been reported that approximately 29.2% of *Clostridium difficile* infection cases that tested positive were of the BI/NAP1/027 type, suggesting significant circulation of this strain in the hospital setting in Cali, Colombia [7].

Information on the epidemiological situation of CDI is scarce in Colombia and Latin America. However, CDI identification is more frequent considering its clinical presentation and the current use of molecular testing with higher availability and improved performance. Thus, Colombian studies have reported prevalence values between 9% and 15% [4,8,9]. However, because these data only refer to certain areas, the purpose of the current study is to describe the epidemiological and clinical characteristics and potential risk factors associated with CDI in a high-complexity center, which manages a high proportion of patients with oncological conditions.

## 2. Materials and Methods

This is an analytical, retrospective, case–control study conducted with a study population in a high-complexity center in the city of Cali, Colombia, during a three-year period from January 2020 to December 2022.

This study expands and adapts the results previously presented in the https://repositorio.usc.edu.co/handle/20.500.12421/5876 (accessed on 28 February 2026), completed at Universidad Santiago de Cali, Colombia.

### 2.1. Patients and Procedures

Our study included patients ≥18 years of age with diarrhea (three or more liquid stools per day) and risk factors for CDI who were registered in the institutional clinical laboratory database with a request for molecular testing with the FilmArray Gastrointestinal (GI) PCR Panel (BioFire Diagnostics; Salt Lake City, UT, USA). This test has a reported sensitivity of ≥94.5% and a specificity of ≥97.1%. Cases were defined as being positive for CD, with a degree of severity and recurrence determined based on the recommendations by the American guidelines for CDI diagnosis and management [10]. The type of exposure to CD was defined according to the following criteria: (1) community-acquired CDI (CA-CDI), which refers to individuals with emerging symptoms within the community or <48 h after hospital admission, or individuals with symptoms that started >12 weeks after their last hospital discharge and (2) hospital-acquired CDI or CDI associated with or facilitated by healthcare (HA-AFHC CDI), defined as occurring >48 h after hospital admission [11]. The neutrophil-to-lymphocyte ratio (NLR) was defined as the ratio between the absolute neutrophil count over the absolute lymphocyte count, using the first complete blood count performed after symptom onset [12,13].

Considering that SARS-CoV-2 infection could have altered the dynamics of CDI—with hygiene measures reducing overall incidence but increasing risk and mortality in severely ill patients receiving antibiotics and admitted to the ICU—the analysis was adjusted for the presence or absence of COVID-19 to avoid confounding bias.

**Figure 1:**
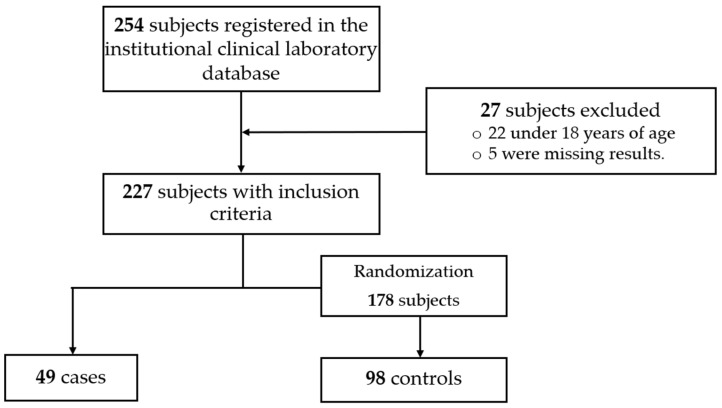
Flowchart of study participant selection.

Patients referred from other hospital institutions were excluded to avoid bias when defining exposure. Considering the number of cases and to increase statistical power, controls were randomly selected from the same database at a 2:1 ratio by age and sex and corresponded to individuals negative for CD (Figure 1).

### 2.2. Data Analysis

For both the general population and cases and controls, we conducted a descriptive analysis of variables according to their nature. Categorical data were analyzed using frequency distribution and relative and absolute frequencies, while quantitative data were analyzed using measures of central tendency (mean and median) and dispersion (standard deviation and interquartile range). Data normality was determined using Kolmogorov–Smirnov test, and to estimate differences in mean, median, and proportion values between cases and controls, Student’s *t*-test, Mann–Whitney U test, and chi-square test were used, respectively, with their corresponding *p*-values. Values < 0.05 were considered statistically significant.

For the multivariate logistic regression model, independent variables with *p* ≤ 0.25 identified in the univariate model were used to identify risk factors associated with CDI in the study population. All values were estimated with a confidence and significance level of 95% and 5%, respectively. Finally, the Hosmer–Lemeshow goodness-of-fit test was conducted. The data were analyzed using the STATA 17.0^®^ statistical software (StataCorp, College Station, TX, USA).

## 3. Results

### 3.1. Demographic and Clinical Characteristics

During the study, 227 molecular tests were requested for participants meeting the inclusion criteria. Consequently, CDI diagnosis was confirmed in 49 cases, 74% of which had been diagnosed in the last study year (patient selection is shown in Figure 1). The incidence rate per admission during the observation period was 2.1 cases per 1000 hospital admissions. Distribution by sex was similar, and participants’ mean age was 57 years; one third of participants had been admitted because of gastroenteric symptoms. Participants’ demographic data, history, and admission diagnoses are described in Table 1. Regarding clinical history, arterial hypertension was more frequent in patients with CDI (55.1% vs. 37.8% in controls; *p* = 0.046). Other clinical histories, such as cancer, cardiovascular disease, diabetes mellitus, or previous COVID-19 infection, showed no significant differences. Hospitalization within the last 90 days was more common among cases (73.5% vs. 58.2%), showing a trend toward statistical significance (*p* = 0.070).

**Figure 2:**
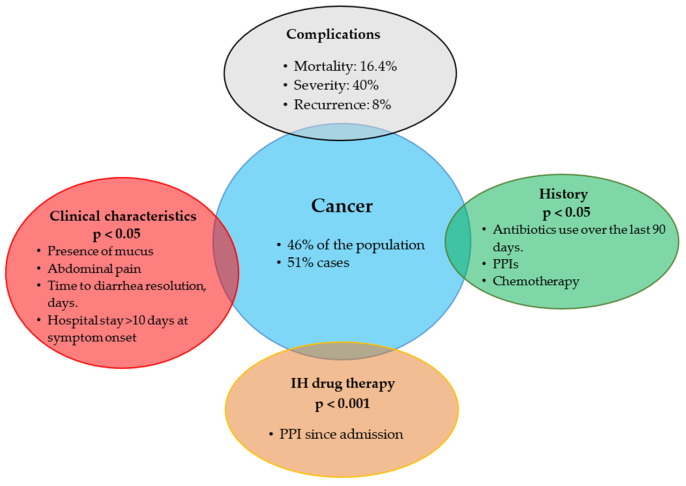
Clinical characteristics of the oncology population. Abbreviations: PPI, proton pump inhibitor.

Patients with cancer accounted for 46% of the population, with no significant differences between both groups. Figure 2 describes some important characteristics of this population, including the clinical variables with the greatest statistical significance and their respective *p*-value in the oncology population.

With the performance of the molecular testing, a CDI prevalence of 21.6% was documented and, in terms of type of exposure, 55% of cases met the criteria for HA-AFHC CDI.

Furthermore, 30-day mortality was 15% with no differences between cases and controls, 18% in patients who met the criteria for CDI severity and 16% in patients with cancer, with no statistically significant differences.

Regarding the clinical manifestations, 55% of participants presented fever at the time of diagnosis, whereas abdominal pain was the most frequent symptom. Abdominal pain, together with the time of resolution of gastroenteric symptoms, were statistically significant findings.

### 3.2. Pharmacological Aspects and Complications

Case–control comparisons showed that antibiotic use in the last 90 days and antibiotic use for >7 days during hospitalization prior to symptom onset showed statistically significant results. Meropenem and piperacillin/tazobactam were the most frequently used antibiotics, as was PPI use since hospital admission. Table 2 shows additional clinical, pharmacological, and laboratory characteristics.

CDI-associated complications—such as severity—were observed in 45% of cases, whereas recurrence of the infection was present in 8% of subjects, who required a new therapeutic regimen. Additionally, imaging signs of colitis and signs of lower gastrointestinal bleeding were observed in 8% and 6% of cases, respectively.

Ninety-six percent of participants received oral vancomycin, of which 80% completed the in-hospital treatment regimen.

### 3.3. Clinical Laboratory and Microbiology Findings

Molecular testing identified microorganisms other than CD in 25% of the subjects, with no statistically significant differences between cases and controls (26.5% vs. 23.5%, *p* = 0.165). The agents detected are shown in Table 3.

Although the mean creatinine level was slightly higher in the control group, it was not statistically significant in patients with severe CDI (*p* = 0.126).

As for inflammatory markers, the average NLR was 11 with no difference between cases and controls. Similarly, no significant differences were found in the PCR results.

### 3.4. Risk Factors

Considering the univariate analysis and its statistical significance, the logistic regression model included the following variables: age >65 years, hospital stay at symptom onset >10 days, other infections diagnosed upon admission, history of cancer, history of diabetes mellitus, history of hospitalization over the last 90 days, antibiotic use over the last 90 days, in-hospital antibiotic use prior to symptom onset >7 days, history of PPI use since hospital admission, and history of outpatient steroid use. In addition, the model was adjusted for other theoretically relevant confounding factors such as sex, history of COVID-19, and steroid use. This model allows us to explain the risk factors for CDI by 34% (Pseudo R^2^).

Considering the clinical and theoretical aspects—and the variation in crude and adjusted odds ratios (ORs)—possible effect modifiers were evaluated; however, no significant differences were found for any of the variables nor additional contributions that could explain such variability.

When evaluating final model fit, adequate data fit (Hosmer–Lemeshow test) was evidenced with a chi-square value of 62.8 (*p* < 0.001). Regarding the model’s classification capacity, it was able to adequately classify 88% of cases (sensitivity) and 73% of controls (specificity); thus, it can adequately rule out factors not associated with CDI. Therefore, we may infer that the likelihood of being a case among the model predictions is 78%, while the likelihood of being a control among the patients that did not have risk factors according to the model was 92%. The results of the model are presented in Table 4.

It is important to highlight that variables were selected for the multivariate model using the standard criterion of a *p*-value < 0.25 in the univariate analysis (Hosmer–Lemeshow), to avoid prematurely excluding potential predictors. Clinically relevant factors identified in the literature—such as age, recent hospitalization, antibiotic exposure, immunosuppression, and proton pump inhibitor use—were also retained to minimize residual confounding

## 4. Discussion

Over the years, CD has become one of the main causes of nosocomial diarrhea and has been associated with the use of antibiotics in up to 30% of cases [14]. We conducted a retrospective study to analyze CDI behavior during the last 3 years considering that it is highly variable.

Information on this topic is scarce in Colombia and Latin America, and prevalence values between 2% and 40% have been reported, interpreted as the number of CDI cases per 100 stool samples. However, prevalence depends on multiple factors such as the population studied, the number of patients included, and the type of study [3,15,16].

In addition to the growing but scarce information available, our study identified a prevalence of 22%, which is slightly higher than the values between 9% and 15% reported in Colombia. However, this figure only reflects the situation of certain health care centers in the main cities of the country. In addition, our study includes a higher number of cases than previous studies [8,17,18]. Furthermore, our findings yielded a prevalence rate of 21 per every 10,000 patients, which was higher than the one reported by another study conducted in the same city [8].

Our study identified no hypervirulent strains, whose circulation has been described in the literature as a predictor of worse outcomes—severity, recurrence, and mortality. Values reported globally for these strains vary from 2% to >60% [19], whereas they have been reported in 11–21% of confirmed cases in Colombia. Because of the scarce data available, these outcomes are also irregular and even contradictory; thus, it is not possible to identify specific risk factors for this event other than those already recognized for infection in general [17,20,21]. Ribotyping provides a reliable tool for strain differentiation and for analyzing genetic relatedness through the identification of variations in ribosomal genes. Its use enhances the discriminatory power of the study, complements phenotypic methods, and allows more precise epidemiological linkage. Overall, this technique strengthens the characterization of isolates and adds greater robustness to the interpretation of the results.

Patient-specific risk factors—such as being older than 65 years and having comorbidities (including cardiovascular, oncologic, and renal) increase the likelihood of CDI and are influenced by additional factors such as greater exposure to medications (mainly antibiotics and PPIs), prior hospitalizations, and prolonged hospital stays [6,8,16]. In our study, the average age was 57 years, while 40% of individuals had a history of cancer, with no significant differences between the groups compared.

Exposure to antibiotics in the last 90 days, hospital stay longer than 10 days, and in-hospital use of PPIs were identified as risk factors with OR values > 1, which is consistent with the findings reported worldwide [9,22,23].

The likelihood of exposure to CDI risk factors is higher among patients with cancer, although there is no causal relationship between the events. Therefore, the disease may progress in a way similar to that in the general population based on what has been described in the literature [24,25]. In our study, antibiotic exposure was not statistically significant, which was surprising; however, this was not the case for the use of PPIs since hospital admission.

The use of antibiotics in this population mostly depended on the reason behind hospital admission, with infectious causes accounting for 10–15% of cases. In addition, other reasons for admission related to neoplasm (solid vs. hematologic), such as pain management, were more frequent [26]. Moreover, other causes of diarrhea should be considered, including those secondary to radiation or chemotherapy, which highlights the need to individualize diagnostic suspicion and conduct targeted studies in high-risk populations such as the one in our study [27].

Among the antibiotics used prior to infection and considered as risk factors, beta-lactam antibiotics are one of the most frequent in up to 60% of cases. However, antibiotic type and spectrum vary according to the presence of other conditions and clinical complications, as well as the site of infection and the level of hospital care [8,16,28]. In our study, meropenem was the most frequently used antibiotic, both within the preceding 90 days and during hospitalization. This is consistent with what has been reported in the literature and is partially explained by their spectrum and their high impact on the disruption of intestinal flora [29]. Although piperacillin–tazobactam was used less frequently in the case group than in the control group, these findings could be explained by differences in the clinical indications for antibiotic therapy between the groups. Scores such as DASC (days of antibiotic spectrum coverage) have been designed, which assess the risk of CDI recurrence using an index that combines the spectrum of antibiotics and the duration of administration rather than the type of antibiotic [30].

The studies consistently demonstrate the strong association between antibiotic use and the development of *Clostridioides difficile* infection in hospitalized patients. The former, through a meta-analysis of patients with antibiotic-associated diarrhea, reported that approximately one-third had CDI, with clindamycin, fluoroquinolones, and cephalosporins being the most frequently implicated antibiotics. In the latter retrospective analysis, a significant correlation was identified between increased institutional consumption of broad-spectrum antibiotics including cephalosporins, fluoroquinolones, and β-lactams and a rise in hospital-onset CDI cases. Collectively, these findings reinforce that recent exposure to broad-spectrum antibiotics is a key determinant of CDI risk and underscore the importance of strengthening antimicrobial stewardship policies to promote rational antibiotic use [31,32].

Recent studies suggest that the risk of CDI increases with an elevation in hospital stay. Similarly, CDI can increase hospital stay after diagnosis even by >15 days, which increases health care costs [19,23]. Consistent with what has been reported in the literature, in our study, the average hospital stay of infected patients prior to symptom onset and after diagnosis was significantly higher, and we found hospital stay longer than 10 days before symptom onset to be a significant risk factor. During the COVID-19 pandemic, some studies showed a higher incidence of CDI compared to the incidence in hospitalized individuals before the epidemic. Increased use of antibiotics and longer hospital stays appear to be contributing factors [33].

These findings correlate with the fact that the most frequent type of infection is HA-AFHC CDI, accounting for 50–80% of cases, although CA-CDI has increased in the last few years and has reached 7–19% [11,18,34]. Consistent with this, our findings were similar to those described in the literature, accounting for 55% and 6%, respectively.

Clinical manifestations vary and can range from asymptomatic to fulminant affections; thus, a clinical approach to some of the most frequent symptoms and the risk factors mentioned above increase diagnostic probability [5,10]. Previous studies have frequently reported abdominal pain and fever, as well as dysenteric stools in patients with CDI [8,9,15,35]. Our study evidenced similar clinical findings, highlighting the presence of abdominal distention and mucus in stools, which were statistically significant.

CDI severity also varies depending on factors such as immune status, antibiotic exposure time, hospital stay, type of strain, and even the definition of the event. It can reach frequencies ranging from 10% to 55%; however, the impact on 30-day mortality was not directly associated with severity and was <10% [8,9,35,36,37]. Our study found a severity of 45% based on the definition used, with no significant differences in terms of mortality in non-severe cases or controls. However, severity was slightly higher (15%) than that reported by previous studies, which could be explained by other variables such as an overall average hospital stay longer than 35 days.

Recurrence of infection (return of CDI symptoms after complete clinical resolution following initial treatment, usually within 8 weeks) behaves in a similar way as severity and can occur in up to 30% of cases after the first episode [10]. In this context, time to diarrhea resolution, as well as persistence of other symptoms including lower gastrointestinal bleeding or imaging findings of colitis, should raise suspicion of recurrence or help rule out other possible causes and complications [34,35,38]. In our study, the time to clinical picture resolution was significantly longer during CDI and symptoms persisted in 21% of cases, without significant differences. The persistent symptoms are defined as the continuation of clinical signs of CDI—diarrhea, abdominal pain, or fever- despite completion of the initial treatment course, without a symptom-free interval. Consequently, the recurrence of hospital-acquired infection after adequate treatment was 8%. Some factors associated with the risk of developing CDI in already colonized individuals have been identified, including admission to the ICU, neoplasia, cirrhosis, hospitalization in the last 6 months, and use of antibiotics in the last 3 months, among others [39].

Similarly, our study reported the presence of other infectious agents in 25% of cases (80% of them being *Escherichia coli*), which is consistent with the findings of several reviews that reported values from 30% to 60%. Whether they behave as colonization or coinfection is still unclear; however, this fact could change the clinical course of the disease [40,41].

Regarding concomitant infection with SARS-CoV-2, available evidence indicates that *Clostridioides difficile* coinfection in hospitalized patients contributes to poorer clinical outcomes, the studies by Maslennikov et al. (2022) and Deda et al. (2023) consistently indicate that coinfection with *Clostridioides difficile* in hospitalized patients with COVID-19 is associated with worse clinical outcomes [42,43]. Both investigations including a retrospective analysis of 809 patients and a nationwide evaluation in the United States demonstrated that the presence of *C. difficile* significantly increases mortality, length of hospital stay, need for critical care, and associated healthcare costs. Collectively, this evidence underscores the clinical and epidemiological importance of surveillance and prevention of *C. difficile* coinfection in the context of SARS-CoV-2 infection. In our study, COVID-19 was observed at a relatively low frequency (21.4%), with no differences between cases and controls. Nevertheless, this subgroup exhibited higher mortality, longer hospital stays, and greater ICU requirements, although these differences were not statistically significant when compared with patients without a COVID-19 diagnosis.

To determine the systemic involvement of CDI, it is important to identify new markers of inflammation and assess those already known, including the presence of leukocytosis, CRP, and kidney function [10,12,44]. Our findings suggest that the CRP tended to be higher in the cases, but the difference was not statistically significant. Regarding the NLR, no differences were observed between groups. Although NLR values were elevated in both CDI cases and individuals without CDI, no significant differences were observed between the groups; but, we believe that further studies are required to determine the prognostic value of this marker.

Early identification of *Clostridioides difficile* infection should be based on a high index of clinical suspicion in hospitalized patients, particularly those with prolonged hospital stays, advanced age, significant comorbidities, and recent exposure to broad-spectrum antibiotics or proton pump inhibitors. The onset of new or persistent diarrhea, especially when accompanied by abdominal distension, mucus in stools, or fever, should prompt timely diagnostic evaluation, particularly in high-risk populations such as patients with cancer, in whom symptoms may be erroneously attributed to other causes.

One of the strengths of our study is that it is one of the studies with the highest number of cases included in Colombia, and it also includes a heterogeneous population like that of any other high-complexity institution. This allows for a comparative analysis of the literature at a national level and a description of a high-risk group such as patients with cancer. Another strength is that measuring factors such as NLR, which suggests a severe inflammatory process, opens the door for further research on this topic.

Our study had some limitations such as its retrospective nature, which increases the possibility of undiagnosed mild or self-limited cases. Similarly, our definition of severity is based purely on laboratory parameters; thus, the frequency of this event could have been overestimated. The integration of patients’ clinical findings with molecular detection using the FilmArray GI PCR Panel allowed for the identification of *Clostridioides difficile* cases with high sensitivity. However, some positive results may correspond to asymptomatic colonization, indicating a risk of overdiagnosis. Therefore, the interpretation of molecular results should always be performed in the context of the clinical presentation to ensure a more accurate diagnosis and avoid unnecessary interventions. Additionally, the use of algorithms incorporating GDH (glutamate dehydrogenase) antigen and A/B toxin by enzyme immunoassay (EIA) is recommended as a complementary diagnostic strategy.

In the present study, the logistic regression model yielded a pseudo-R^2^ of approximately 34%, a value consistent with the multifactorial nature of the outcome and the explanatory aim of case–control studies. The wide confidence intervals observed for some odds ratios likely reflect a limited number of events, low-frequency exposures, and clinical heterogeneity, rather than model misspecification. Adjustment for clinically relevant covariates entailed an expected reduction in precision, strengthening internal validity by improving control of confounding. Overall, the model demonstrates an appropriate balance between model fit, clinical plausibility, and generalizability, supporting the robustness of the observed associations.

## 5. Conclusions

CDI may involve a wide range of clinical manifestations; thus, its underdiagnosis should be avoided. Consequently, identifying risk factors for CDI becomes necessary, particularly among patients with nosocomial diarrhea and special populations, with factors such as a hospital stay longer than 10 days prior to symptom onset and in-hospital exposure to PPIs and antibiotics over the last 90 days being significant, as evidenced in our study. Finally, a longer hospital stay results in a higher risk of complications and most likely in increased health care costs.

## Figures and Tables

**Table 1:** Participants’ sociodemographic characteristics and history.

Variables	Total	Cases	Controls	*p*-Value
	*n* = 147	*n* = 49	*n* = 98	
**Characteristics**				
Women	74 (50.3)	25 (51.1)	49 (50)	0.907
Average age (SD) in years	57.5 (17.9)	60.1 (15.1)	56.2 (19.1)	0.180
<45 years	35 (23.8)	8 (16.3)	27 (27.6)	0.132
45–54 years	18 (12.2)	8 (16.3)	10 (10.2)	0.286
55–64 years	39 (26.5)	16(32.7)	23 (23.5)	0.234
>65 years	55 (37.4)	17 (34.7)	38 (38.8)	0.630
Mortality <30 days	23 (15.6)	8 (16.3)	15 (15.3)	0.870
Contributory health insurance system	102 (69.4)	31 (63.3)	71 (72.4)	0.255
**Clinical history**				
Arterial hypertension	64 (43.5)	27 (55.1)	37 (37.8)	0.046
Cancer	67 (45.6)	25 (51)	42 (42.9)	0.349
Cardiovascular disease (ischemic heart disease, heart failure, and cerebrovascular disease)	30 (20.4)	11 (22.4)	19 (19.4)	0.664
Diabetes mellitus	36 (24.5)	8 (16.3)	28 (28.6)	0.104
COVID-19	29 (19.7)	8 (16.3)	21 (21.4)	0.464
Hospitalization over the last 90 days	93 (63.3)	36 (73.5)	57 (58.2)	0.070
Others	36 (24.5)	15 (30.6)	21 (21.4)	0.222
**Pharmacological History**				
Antibiotic used in the last 90 days	53 (36.1)	26 (53.1)	27 (27.6)	0.002
Meropenem	16 (10.9)	10 (38.5)	6 (6.1)	0.009
IV vancomycin	16 (10.9)	8 (30.8)	8 (8.2)	0.134
Piperacillin/tazobactam	13 (8.8)	6 (23.1)	7 (7.1)	0.304
Cefepime	13 (8.8)	6 (23.1)	7 (7.1)	0.304
Ampicillin/sulbactam	6 (4.1)	4 (15.4)	2 (2)	0.077
Clindamycin	3 (2)	3 (11.5)	0	-
Ceftriaxone	5 (3.4)	3 (11.5)	2 (2)	0.198
Others	23 (15.6)	10 (38.5)	13 (13.3)	0.261
PPIs	86 (58.5)	28 (57.1)	58 (59.2)	0.813
Chemotherapy	51 (34.7)	17 (34.7)	34 (34.7)	1.000
Steroids	33 (22.4)	7 (14.3)	26 (26.5)	0.093
**Diagnosis upon admission**				
Gastroenteritis	46 (31.3)	16 (32.7)	30 (30.6)	0.801
Other infections (UTI, ST, FN)	29 (19.7)	13 (26.5)	16 (16.3)	0.143
Other diagnoses	72 (49)	20 (40.8)	52 (53.1)	0.162
**Unit that identified the symptoms**				
Emergency unit	73 (49.7)	23 (46.9)	50 (51)	0.641
Hospitalization unit	56 (38.1)	21 (42.9)	35 (35.7)	0.401
ICU	18 (12.2)	5 (10.2)	13 (13.3)	0.594

Abbreviatures: SD: standard deviation; IV: intravenous; PPI: pump proton inhibitor; UTI: urinary tract infection; FN: febrile neutropenia; ST: soft tissues; ICU: intensive care unit.

**Table 2:** Clinical, pharmacological, and laboratory characteristics and complications.

Variables	Total	Cases	Controls	*p*-Value
	*n* = 147	*n* = 49	*n* = 98	
**Clinical characteristics (%)**				
Bowel movement frequency, average (SD)	5 (1.6)	5 (1.8)	5 (1.6)	1.000
Presence of mucus	43 (29.3)	24 (49)	19 (19.4)	<0.001
Presence of blood	13 (8.8)	7 (14.3)	6 (6.1)	0.100
Abdominal pain	101 (68.7)	44 (89.8)	57 (58.2)	<0.001
Abdominal distension	75 (51)	31 (63.3)	44 (44.9)	0.036
Vomiting	19 (12.9)	8 (16.3)	11 (11.2)	0.385
Fever	82 (55.8)	23 (46.9)	59 (60.2)	0.127
Diarrhea resolution in days, average (SD)	4.6 (2.8)	5.9 (2.4)	3.9 (2.7)	<0.001
Hospital stay, days. Average (SD)	39.1 (34.0)	47.7 (34.5)	35.3 (33.0)	0.040
Hospital stay >10 days at symptom onset	69 (46.9)	33 (67.3)	36 (36.7)	<0.001
Hospital stay 30 days after diagnosis. Average (SD)	11.9 (7.9)	15.1 (7.3)	10.4 (7.9)	0.001
**In-hospital drug therapy (%)**				
Use of IH Atb. before symptom onset	94 (63.9)	33 (67.3)	61 (62.2)	0.540
Use of IH Atb. before symptom onset >7 days	51 (34.7)	23 (46.9)	28 (28.6)	0.027
Meropenem	50 (34)	14 (42.4)	36 (36.7)	0.325
Cefepime	39 (26.5)	13 (39.4)	26 (26.5)	1.000
IV vancomycin	46 (31.3)	12 (36.4)	34 (34.7)	0.208
Piperacillin/tazobactam	47 (32)	9 (27.3)	38 (38.8)	0.012
Metronidazole	7 (4.8)	4 (12.1)	3 (3.1)	0.171
Others	50 (34)	13 (39.4)	37 (37.8)	0.176
PPI since hospital admission	76 (51.7)	44 (89.8)	32 (32.7)	<0.001
Loperamide	106 (72.1)	34 (69.4)	72 (73.5)	0.603
IH chemotherapy	55 (37.4)	20 (40.8)	35 (35.7)	0.547
Lactulose	26 (17.7)	7 (14.3)	19 (19.4)	0.445
PEG	17 (11.6)	5 (10.2)	12 (12.2)	0.715
Bisacodyl	21 (14.3)	3 (6.1)	18 (18.4)	0.046
**CD-associated complications (%)**				
Infection severity	-	22 (44.9)	-	-
Infection recurrence	-	4 (8.2)	-	-
Symptom persistence	31 (21.1)	7 (14.3)	24 (24.5)	0.153
Lower GI bleeding	3 (2)	3 (6.1)	0	-
Colitis by TAC	6 (4.1)	4 (8.2)	2 (2)	0.077
**Paraclinical test values upon diagnosis**				
Leukocytes, median (IQR)	8890 (10,280)	10,340 (11,730)	8460 (8380)	0.114
Neutrophils, median (IQR)	5930 (9250)	7640 (10,590)	5665 (7816)	0.118
Lymphocytes, median (IQR)	1010 (1260)	970 (800)	1010 (1350)	0.531
NLR, average (SD)	11.2 (16.3)	11.5 (11.6)	11.7 (18.3)	0.936
Hemoglobin, average (SD)	10.3 (3.2)	9.9 (4.3)	10.5 (2.5)	0.370
Platelets, average (SD)	255,994.3 (191,131.9)	256,653.5 (189,298.9)	255,664.8 (193,009.8)	0.976
CRP, average (SD)	135.1 (109.1)	160 (97.7)	124.8 (112.7)	0.053
Creatinine, average (SD)	2.2 (5.1)	1.5 (1.2)	2.5 (6.2)	0.126
Other pathogens detected by GI panel (%)	36 (24.5)	13 (26.5)	23 (23.5)	0.684

Abbreviatures: Atb: antibiotics; CD: *Clostridioides difficile*; SD: standard deviation; IV: intravenous; GI: gastrointestinal; PPI: proton pump inhibitor; IH: in-hospital; PEG: polyethylene glycol; IQR: interquartile range; NLR: neutrophil/lymphocyte ratio.

**Table 3:** Other pathogens detected by molecular panel.

Coinfection (%)	Case *n* = 49	Controls *n* = 98	Total *n* = 147
Sapoviruses (Genogroups I, II, IV, and V)	1 (2.0)	0 (0.0)	1 (0.7)
Enteropathogenic *Escherichia coli* (EPEC)	4 (8.2)	11 (11.2)	15 (10.2)
Enteroaggregative *Escherichia coli* (EAEC)	4 (8.2)	6 (6.1)	10 (6.8)
Shiga-producing *Escherichia coli* (STEC) stx1/stx2	3 (6.1)	3 (3.1)	6 (4.1)
Enterotoxigenic *Escherichia coli* lt/st	1 (2.0)	0 (0.0)	1 (0.7)
*Escherichia coli* 0157	1 (2.0)	0 (0.0)	1 (0.7)
Norovirus GI/GII	1 (2.0)	1 (1.0)	2 (1.4)
*Campylobacter* (*C. jejuni*/*C. coli*/*C. upsaliensis*)	1 (2.0)	1 (1.0)	2 (1.4)
*Cryptosporidium*	0 (0.0)	1 (1.0)	1 (0.7)

**Table 4:** Adjusted odds ratio (OR) of the logistic regression model for ICD risk factors.

Variable	Adjusted OR	95% CI	*p*-Value
Atb last 90 days	3.3	1.3–8.3	0.01
Hospital stay >10 days at symptom onset	9.74	1.14–83.5	0.01
PPI since hospital admission	20.41	6.91–60.27	<0.001

Abbreviatures: Atb: antibiotics; PPI: proton pump inhibitor; CI: confidence interval.

## Data Availability

The original contributions presented in the study are included in the article, and further inquiries can be directed to the corresponding author.

## Abbreviations

The following abbreviations are used in this manuscript:

CDI	*Clostridioides difficile* infection
PPIs	Proton Pump Inhibitors
NLR	Neutrophil-to-Lymphocyte Ratio
DASC	Days of Antibiotic Spectrum Coverage
